# The new obesity classification based on weight history is not proposed as a guideline

**DOI:** 10.20945/2359-3997000000571

**Published:** 2022-11-28

**Authors:** Bruno Halpern, Marcio C. Mancini, Maria Edna de Melo, Rodrigo N. Lamounier, Rodrigo O. Moreira, Mario K. Carra, Cintia Cercato, Cesar Luiz Boguszewski

**Affiliations:** 1 Hospital 9 de Julho Centro de Obesidade São Paulo SP Brasil Centro de Obesidade, Hospital 9 de Julho, São Paulo, SP, Brasil; 2 Universidade de São Paulo Departamento de Endocrinologia e Metabolismo Grupo de Obesidade e Síndrome Metabólica São Paulo SP Brasil Grupo de Obesidade e Síndrome Metabólica, Departamento de Endocrinologia e Metabolismo, Universidade de São Paulo, São Paulo SP, Brasil; 3 Centro de Diabetes de Belo Horizonte Belo Horizonte MG Brasil Centro de Diabetes de Belo Horizonte, Belo Horizonte, MG, Brasil; 4 Instituto Estadual de Diabetes e Endocrinologia Luiz Capriglione Rio de Janeiro RJ Brasil Instituto Estadual de Diabetes e Endocrinologia Luiz Capriglione, Rio de Janeiro, RJ, Brasil; 5 Universidade de São Paulo Departamento de Endocrinologia Grupo de Diabetes São Paulo SP Brasil Grupo de Diabetes, Departamento de Endocrinologia, Universidade de São Paulo, São Paulo, SP, Brasil; 6 Associação Brasileira para o Estudo da Obesidade e Síndrome Metabólica São Paulo SP Brasil Associação Brasileira para o Estudo da Obesidade e Síndrome Metabólica, São Paulo, SP, Brasil; 7 Serviço de Endocrinologia e Metabologia do Hospital de Clínicas da Universidade Federal do Paraná Departamento de Clínica Médica Curitiba PR Brasil Serviço de Endocrinologia e Metabologia do Hospital de Clínicas da Universidade Federal do Paraná (SEMPR), Departamento de Clínica Médica, Curitiba, PR, Brasil; 8 Sociedade Brasileira de Endocrinologia e Metabolismo Rio de Janeiro RJ Brasil Sociedade Brasileira de Endocrinologia e Metabolismo, Rio de Janeiro, RJ, Brasil

**DEAR EDITOR**

We would like to thank Candido and colleagues for their interest and kind words in regard to the proposed obesity classification based on weight history, as well as for the suggestion for its improvement (1). Indeed, as a new proposal, it certainly could be improved and refined in the medium-to-long term, as we pointed out several times in our manuscript (2).

Importantly, however, the main goal of this classification is to provide a new parameter to evaluate the clinical response to obesity treatment based on maximum weight attained in life (MWAL), a piece of information rarely asked or written in medical records. The classification is not intended as a definitive “guideline”, in which every individual who achieves the threshold for “controlled obesity” does not need to lose more weight. This is clearly stated in the following sentence: “*An individual considered to have controlled obesity could derive benefits from losing further weight or be a candidate for bariatric surgery. Likewise, individuals with reduced (or unchanged) obesity might not need to lose more weight if they have a low overall burden from their high BMI. This classification is rather intended to provide important information for discussion with the patient and, as any other classification, should be considered in the overall context of the patient's health and long-term goals”* (2). Moreover, the classification also includes the percentage of weight loss, so an individual with MWAL of 100 kg and 160 cm (BMI 39 kg/m^2^) who loses 11 kg, would be assigned to class II obesity (10% controlled), while if the same subject loses 20 kg, he/she would be considered as having class II obesity (20% controlled). The presence of the percentage of weight loss when classifying an individual should help clinicians and patients understand the clinical evolution and set personalized goals.

As such, we agree that several individuals with higher BMIs would benefit from further weight loss. However, the majority of guidelines, as the ACC/AHA/TOS, the Brazilian guidelines, and others, recommend a weight loss of 5% to 10% as a target, irrespectively of the initial BMI (3–5). In the European Guideline cited by the authors, it is stated that: *5%-15% weight loss over a period of 6 months is realistic and of proven health benefit {level 1}. A greater (20% or more) weight loss* *MAY BE* *considered for those with greater degrees of obesity (BMI ≥ 35 kg/m*^2^*)* (6). In the Argentinian guideline also cited, a higher weight loss for these individuals (15%-20%) is indeed suggested, but there is no reference in the text as to where this recommendation came from (it is probably a specialist opinion) neither it is discussed whether this is a feasible threshold without bariatric surgery (7).

Moreover, we do have evidence from a post-hoc analysis of the LOOK AHEAD study that individuals who lost 10% of their weight in the first year had 21% lower mortality after almost two decades, and the mean BMI from this cohort before intervention was 36 kg/m^2^ (8).

As such, with these guidelines in context, we put a higher value on weight targets that are clinically feasible for a greater proportion of patients, especially because there is evidence that the mean percentage of weight loss achieved with lifestyle interventions or antiobesity medications is almost the same in individuals with higher or lower BMIs (9).

In regard to the comment about our classification using BMI as a criterium and the absence of comorbidities, this was already cited in our text in the limitations section; as such, the comment just reproduced what we had already mentioned.

We appreciate your remark as it highlights the importance of scientific dialogue, as well as reinforces that this is a classification and not a guideline. Moreover, as we plan to validate this classification in several scenarios, and with new treatments being developed that are able to produce greater weight loss in a higher proportion of individuals (10), our classification could be changed in the future if evidence suggests that other thresholds are more appropriate for defining the terms “reduced” or “controlled' obesity.
